# Co-application of dazomet and azoxystrobin reconstructs soil microbial communities and suppresses the violet root rot of *Codonopsis tangshen* under a continuous cropping system

**DOI:** 10.1128/spectrum.01088-25

**Published:** 2025-07-23

**Authors:** Wuxian Zhou, Xiaofang Wang, Xiaogang Jiang, Darong Li, Meide Zhang, Daye Huang, Jie Guo, Jingmao You, Qingfang Wang

**Affiliations:** 1 Key Laboratory of Biology and Cultivation of Chinese Herbal Medicines, Ministry of Agriculture and Rural Affairs, Institute of Chinese Herbal Medicines, Hubei Academy of Agricultural Sciences 117996, Enshi, Hubei, China; 2 Jiangsu provincial key lab for solid organic waste utilization, Key lab of organic-based fertilizers of China, Jiangsu Collaborative Innovation Center for Solid Organic Wastes, Educational Ministry Engineering Center of Resource-saving fertilizers, Nanjing Agricultural University 70578, Nanjing, China; 3 Hubei Biopesticide Engineering Research Centre 675140 , Wuhan, Hubei, China; USDA-ARS-NPRL, Dawson, Georgia, USA

**Keywords:** continuous cropping obstacle, soil-borne disease, soil fumigation, rhizosphere microbial community, soil property

## Abstract

Violet root rot (VRR) is a devastating soil-borne disease that severely affects *Codonopsis tangshen* (*C. tangshen*) production, with limited effective control strategies available. This study aimed to develop an effective method for controlling VRR and explore its underlying mechanisms. A 2-year field experiment was conducted to investigate the impact of dazomet fumigant alone (D) and in combination with azoxystrobin (DA) on the yield, VRR incidence, medicinal component level, soil biochemical properties, and rhizosphere microbial community structure of continuously cropped *C. tangshen*. The results showed that both D and DA significantly increased the yield by 112.3% and 173.8% in 2019, and by 179.1% and 520.9% in 2020, respectively. Moreover, D and DA significantly reduced the VRR incidence by 87.9% and 100.0% in 2019, and by 14.7% and 100.0% in 2020, respectively. Furthermore, treatments D and DA changed the levels of certain medicinal components, altered partial soil biochemical properties (including pH, enzyme activities, and nutrient levels), and shifted the soil microbial community structure. Notably, the DA treatment significantly decreased the abundance of potential soil-borne pathogen *Helicobasidium*, while increasing the abundance of potential pathogen-suppressive microbes like *Rhodanobacter*, *Saccharimonadales*, *Acidothermus*, and *Tetracladium*, in both 2019 and 2020. The reduced pathogen abundance and restructured microbial community may collectively contribute to the complete suppression of VRR under DA treatment. This study offers an effective strategy for controlling VRR in *C. tangshen* under continuous cropping systems, contributing to the sustainable development of the *C. tangshen* industry.

**IMPORTANCE:**

As a valuable medicinal and edible plant, *Codonopsis tangshen* significantly contributes to rural economies in China’s mountainous areas. However, its cultivation faces severe threats from violet root rot (VRR), a devastating soil-borne disease lacking effective control measures. Our study demonstrated that combined soil fumigation and azoxystrobin application (DA) completely prevented VRR occurrence for 2 years while simultaneously reducing pathogen abundance and restructuring the soil microbial community, which may be key factors in disease suppression. This study provides novel insights into the mechanisms underlying the complete control of VRR by DA treatment and is beneficial for the high-quality development of the *C. tangshen* industry.

## INTRODUCTION

*Codonopsis tangshen* is an important medicinal and edible plant in China, with a cultivation area of about 30,000 hectares and an annual output value of up to 18 billion renminbi (approximately 2.5 billion US dollars). The dried roots of *C. tangshen* are the primary source of Codonopsis Radix, renowned for their therapeutic benefits in treating cough and asthma, strengthening the spleen and stomach, and enhancing hematopoiesis (1, 2). Violet root rot (VRR), a prevalent soil-borne disease caused by *Helicobasidium* pathogens, affects a range of plants, including *Codonopsis tangshen* (3), *Malus domestica* (4), and *Pseudostellaria heterophylla* (5). The disease is characterized by the formation of violet mycelial webbing on the roots, forming a distinctive fungal mat on the surface of plant roots. Farmers commonly refer to this symptom as “red skin disease.” In a continuous cropping system, VRR is particularly prevalent in *C. tangshen*, leading to yield reductions of up to 50.0% in mild cases and complete crop failure in severe cases (6). To combat these diseases and boost yields, farmers often resort to the overuse of fungicides, which not only compromises the quality of Codonopsis Radix but also hampers the sustainable development of the *C. tangshen* industry. Therefore, there is an urgent need for effective strategies to control the VRR and mitigate its impact on the productivity of *C. tangshen*.

Numerous strategies have been recognized for controlling soil-borne diseases, with soil fumigation and fungicide application emerging as especially effective approaches in various agricultural systems (7–9). Dazomet, a broad-spectrum soil fumigant, decomposes into volatile toxic products such as methyl isothiocyanate, formaldehyde, and hydrogen sulfide upon soil application. These volatile compounds penetrate into soil crevices, effectively eradicating pathogenic microorganisms, nematodes, and weed seeds (10). Azoxystrobin, a potent fungicide, is distinguished by its broad-spectrum efficacy, low residue level, and high absorbability (11). It is instrumental in neutralizing soil-borne pathogens and stimulating plant growth, suitable for seed soaking, foliar spray, and soil sterilization (12). However, the applicability and effectiveness of dazomet and azoxystrobin in *C. tangshen* cultivation, specifically for VRR control and plant growth promotion, remain uncertain. Previous studies demonstrated a strong correlation between the prevalence of soil-borne disease and disruptions in the soil microbial community, as well as the proliferation of pathogens in cultivated soil (13, 14). Moreover, soil properties such as pH, enzymatic activity, and nutrient levels play a critical role in regulating microbial reproduction and nutrient cycling (15, 16). Therefore, it is crucial to explore the potential impacts of dazomet and fungicide on the rhizosphere microbial community structure and soil biochemical properties.

In this study, we evaluated the efficacy of dazomet fumigation alone (D) and in combination with azoxystrobin (DA) in controlling VRR in continuously cropped *C. tangshen*. Specifically, we examined (i) the effects of these treatments on the yield, VRR incidence, and medicinal component level of *C. tangshen*; (ii) their impacts on soil biochemical properties and rhizosphere microbial community structure; and (iii) the relationships between yield, VRR incidence, soil biochemical properties, and rhizospheric soil microbial communities and metabolic pathways. This study aims to develop an effective method for controlling VRR in *C. tangshen* and to elucidate its underlying mechanisms, thereby supporting the sustainable development of the *C. tangshen* industry.

## RESULTS

### The growth and disease incidence of *Codonopsis tangshen*

**Fig 1:**
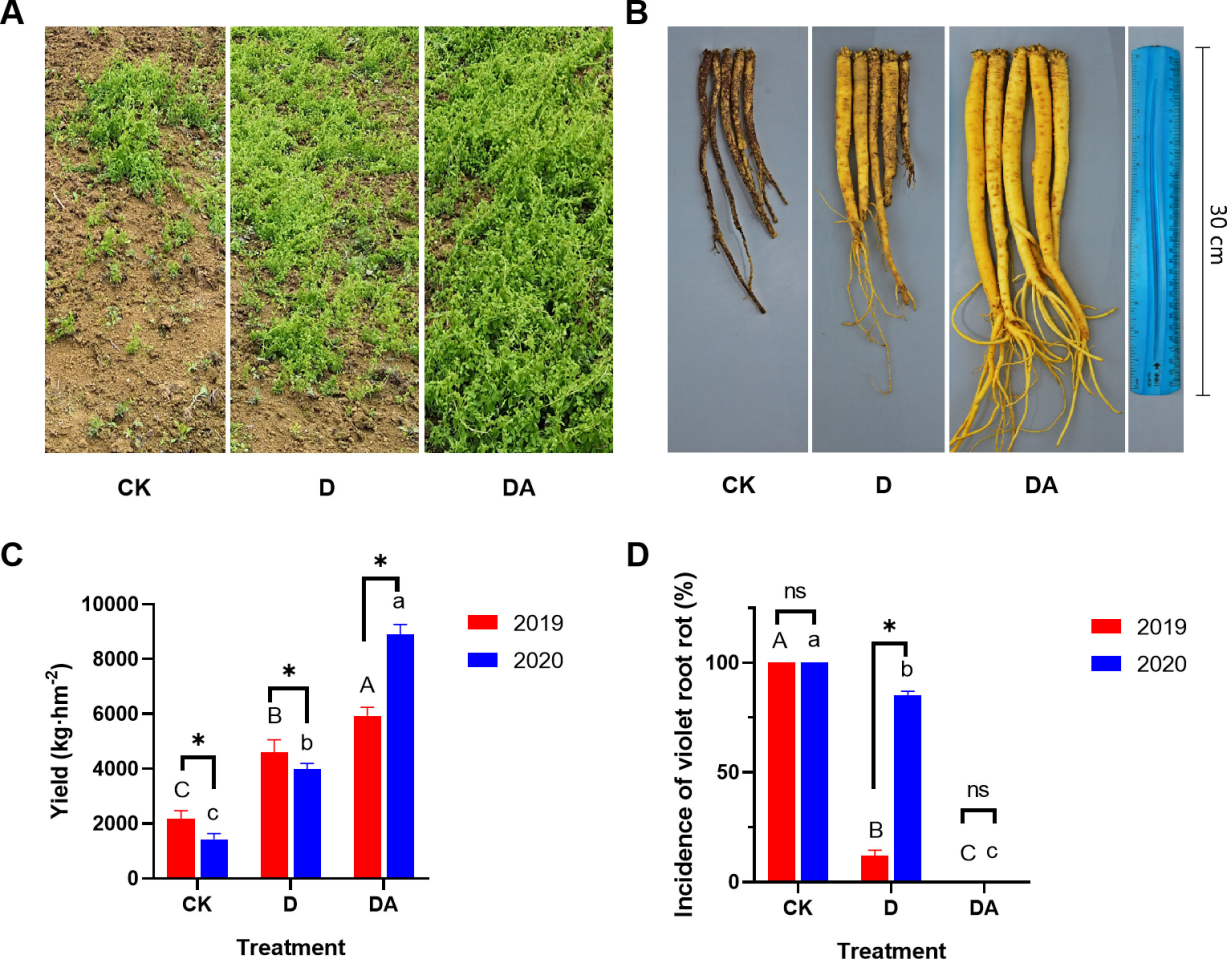
The plant growth (**A**), root features (**B**), yield (**C**), and disease incidence (**D**) of *C. tangshen* in different treatments. Different capital letters indicate significant differences (*P* < 0.05) among treatments in 2019, while different lowercase letters indicate significant differences (*P* < 0.05) among treatments in 2020. “*” and “ns” indicate significant (*P* < 0.05) and non-significant (*P* > 0.05) differences between the same treatments in different years, respectively. Figure 1A shows a recomposed image featuring representative sections extracted from field photographs of CK, D, and DA treatments. Figure 1B displays a rearranged and recombined image derived from the same original photograph.

The treatments D and DA significantly enhanced the growth of *C. tangshen* and reduced the VRR incidence under the continuous cropping system in 2019 (Fig. 1A and B). Compared with the control (CK), both D and DA significantly increased the yield by 112.3% and 173.8% in 2019, and by 179.1% and 520.9% in 2020, respectively (Fig. 1C and D). Moreover, both D and DA significantly decreased the VRR incidence by 87.9% and 100.0% in 2019, and by 14.7% and 100.0% in 2020, respectively. Notably, the DA completely averted the occurrence of VRR across two growing seasons, a result not matched by the D, indicating that DA was a more potent approach for controlling VRR compared with D. Additionally, the yield of the DA in 2020 was significantly higher than that in 2019, in contrast to the declining yields observed in both CK and D. This could be attributed to the higher VRR incidence in CK and D, which led to increased mortality of *C. tangshen*, thereby resulting in lower yield (Fig. 1D).

### The medicinal components in *Codonopsis tangshen*

**Fig 2:**
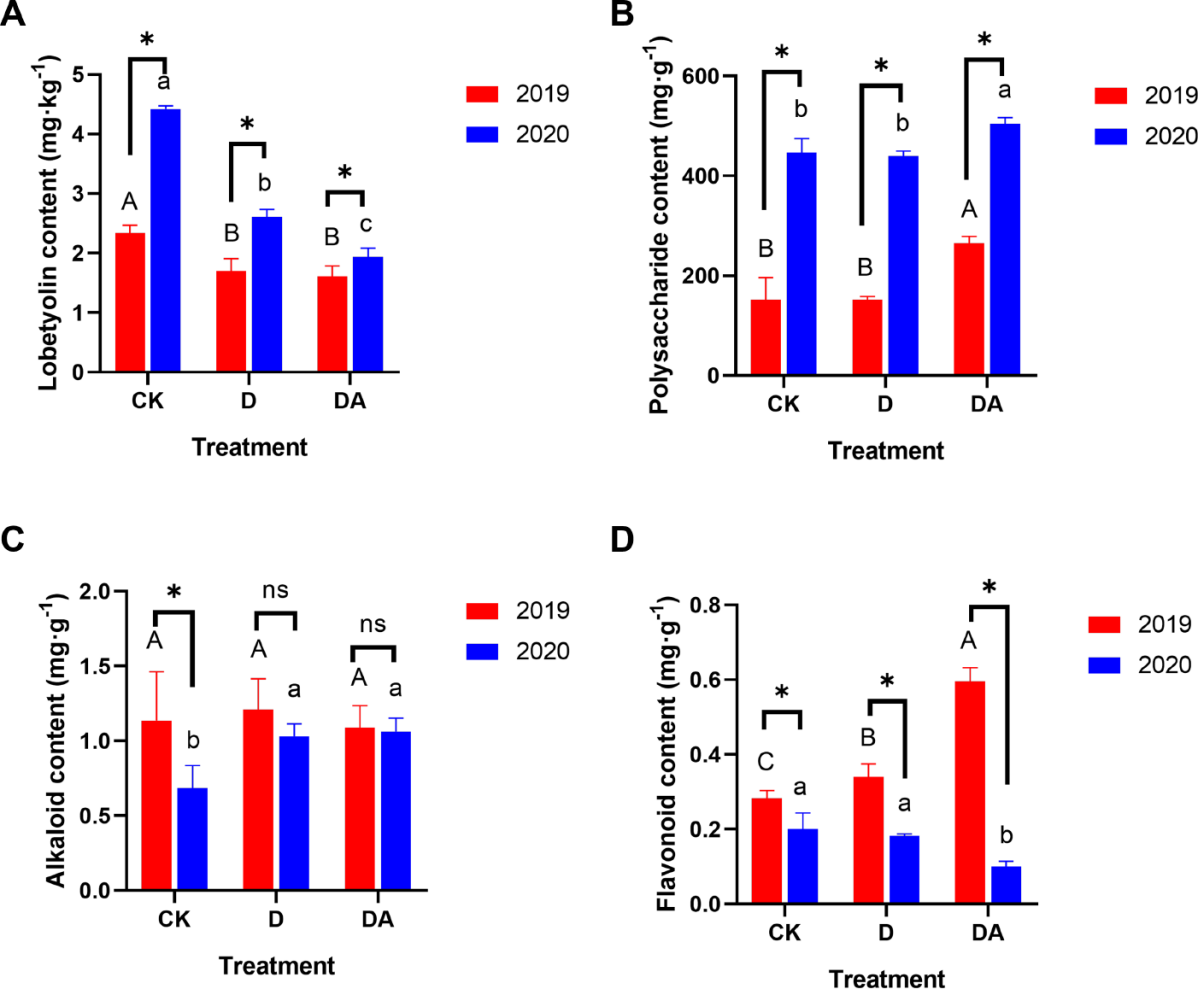
The contents of active components in *C. tangshen* dried root under different treatments. (A) Lobetyolin; (B) polysaccharide; (C) alkaloid; and (D) flavonoid. Different capital letters indicate significant differences (*P* < 0.05) among treatments in 2019, while different lowercase letters indicate significant differences (*P* < 0.05) among treatments in 2020. “*” and “ns” indicate significant (*P* < 0.05) and non-significant (*P* > 0.05) differences between the same treatments in different years, respectively.

A comparative analysis of medicinal components such as lobetyolin, polysaccharide, alkaloid, and flavonoid was conducted among different treatments. The lobetyolin content in both D and DA was significantly lower than that in the CK for both years, 2019 and 2020 (Fig. 2A). In contrast, the DA consistently exhibited a significantly higher polysaccharide content compared with CK and D over the two consecutive years (Fig. 2B). Notably, in 2020, the alkaloid content in both D and DA increased significantly by 50.6% and 55.2%, respectively, compared with CK (Fig. 2C). However, no significant differences were observed among the treatments in 2019, suggesting a time-dependent accumulation of alkaloids in response to D and DA. Furthermore, the flavonoid content exhibited a complex metabolic pattern, with the DA showing a marked increase in 2019 but a significant decrease in 2020 when compared with the CK (Fig. 2D).

### Rhizosphere soil properties

**Fig 3:**
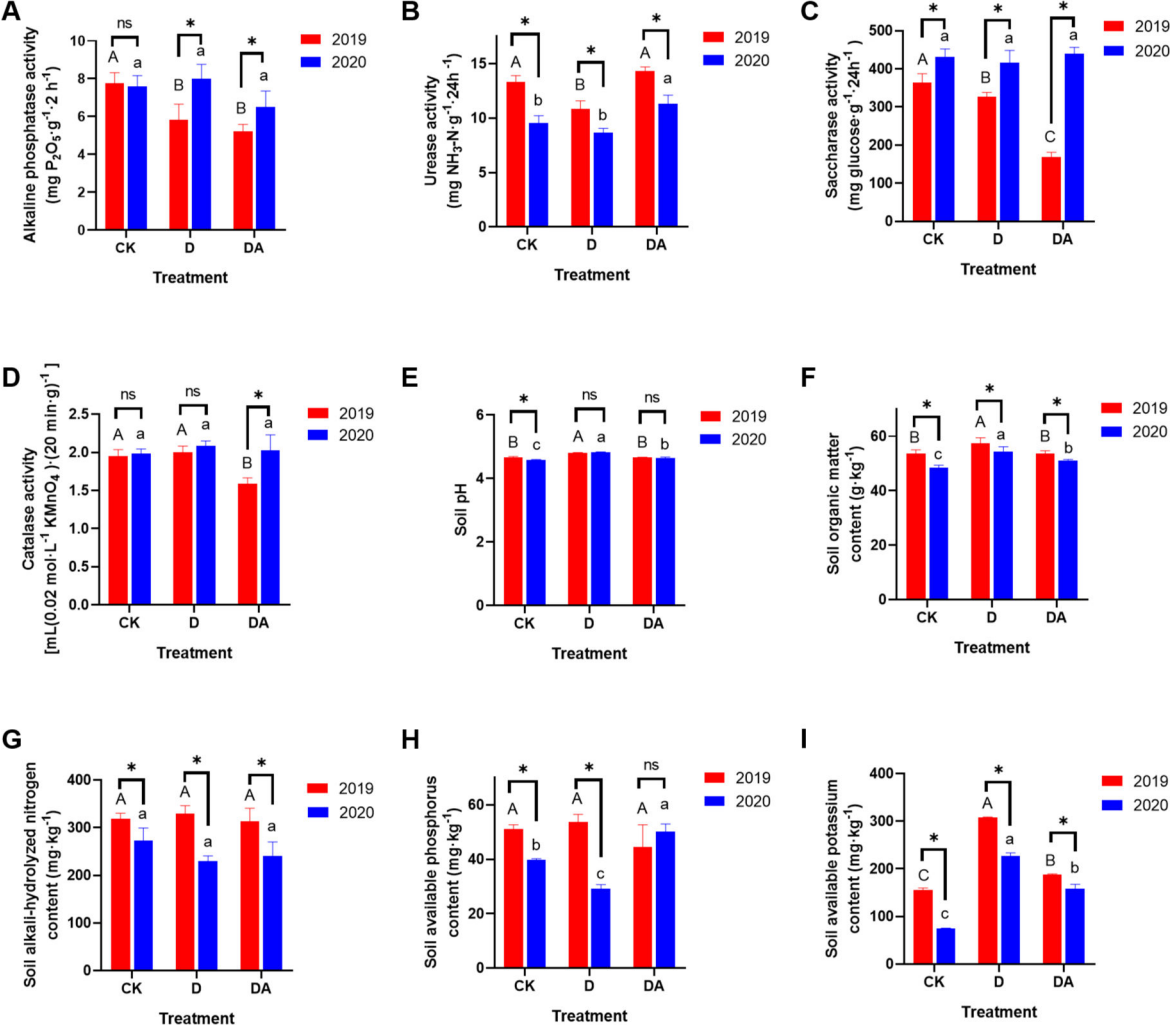
The soil properties of *C. tangshen* across different treatments. (A) Alkaline phosphatase activity; (B) urease activity; (C) saccharase activity; (D) catalase activity; (E) soil pH; (F) soil organic matter; (G) soil alkali-hydrolyzed nitrogen; (H) soil available phosphorus; and (I) soil available potassium. Different capital letters indicate significant differences (*P* < 0.05) among treatments in 2019, while different lowercase letters indicate significant differences (*P* < 0.05) among treatments in 2020. “*” and “ns” indicate significant (*P* < 0.05) and non-significant (*P* > 0.05) differences between the same treatments in different years, respectively.

The D and DA exerted noticeable effects on soil properties (Fig. 3). In 2019, both D and DA significantly reduced the activities of alkaline phosphatase and saccharase, with no significant impact observed in 2020. Notably, the DA substantially increased the urease activity in 2020, contrasting with the negligible effect in 2019. Additionally, the DA led to a significant decrease in catalase activity in 2019, with no notable effect in 2020.

Notable differences in soil pH, organic matter content, alkali-hydrolyzed nitrogen, available phosphorus, and available potassium levels were detected among different treatments (Fig. 3). The D significantly improved the soil pH and organic matter level in both 2019 and 2020. In 2020, the DA not only increased the soil pH and organic matter level but also notably increased the available phosphorus level. Moreover, both D and DA significantly increased the content of soil available potassium in 2019 and 2020. These results indicated that both soil fumigation alone and in combination with azoxystrobin could change partial soil biochemical properties.

### Alpha and beta diversities

**Fig 4:**
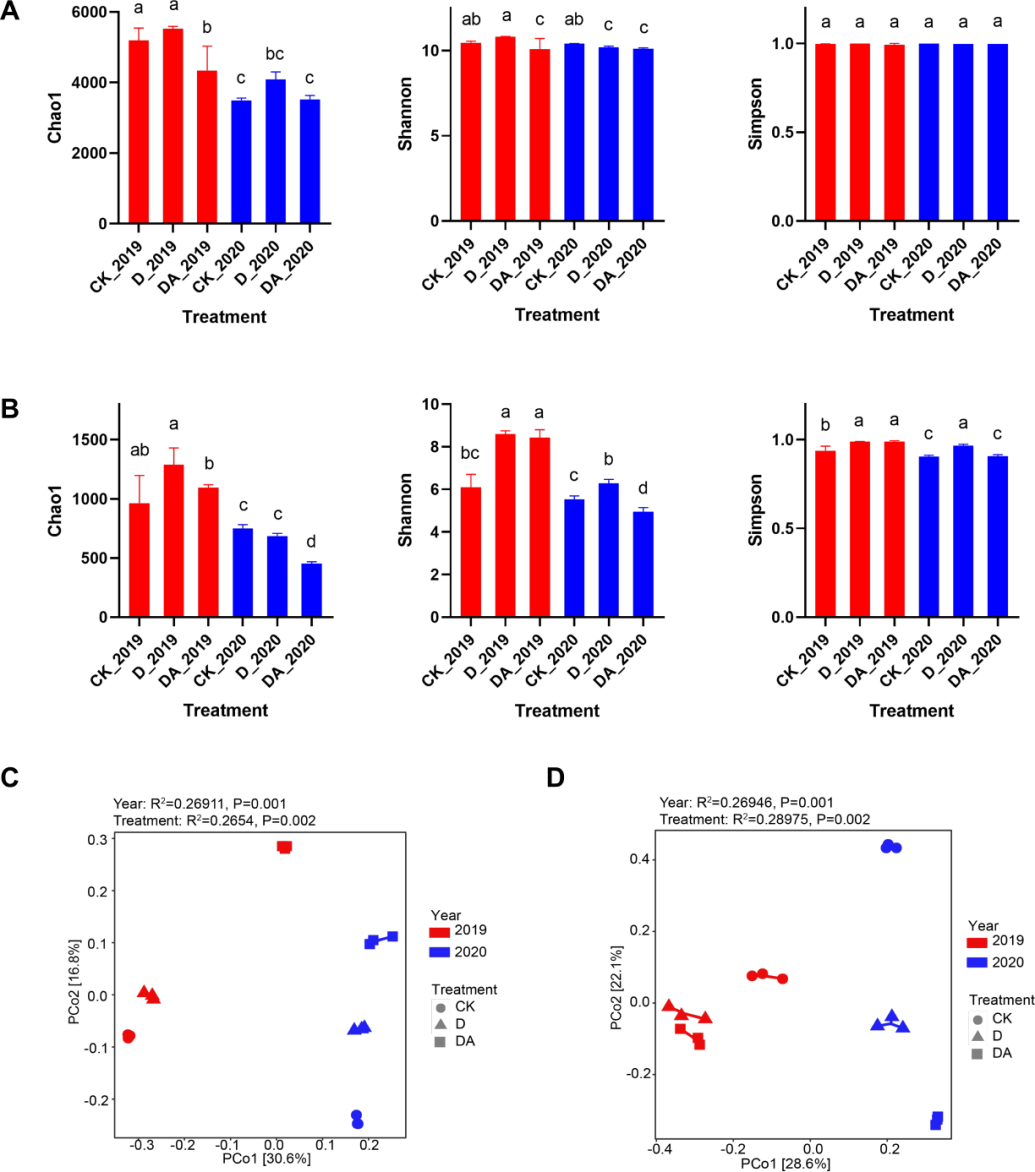
Alpha diversity indices and principal coordinate analysis (PCoA) of the microbial communities in the rhizosphere soil of *C. tangshen*. (A) Bacterial alpha diversity indices; (B) fungal alpha diversity indices; (C) bacterial principal coordinate analysis; and (D) fungal principal coordinate analysis. Different capital letters indicate significant differences (*P* < 0.05) among treatments in 2019, while different lowercase letters indicate significant differences (*P* < 0.05) among treatments in 2020.

The microbial diversity and richness of the rhizosphere soil were assessed using alpha (α)-diversity indices such as Chao1, Shannon, and Simpson (Fig. 4A and B). In 2019, the D significantly increased the bacterial α-diversity, like Chao and Shannon. However, in 2020, the Chao1 index continued to show a significant increase, whereas indices like Shannon and Simpson showed a decline. Conversely, the DA remarkably reduced Chao1 in 2019, but had no significant impact in 2020. In terms of fungal α-diversity, both D and DA significantly increased all measured fungal α-diversity indices in 2019. However, the DA significantly decreased the Chao1 index in 2020.

Beta (β)-diversity analysis was conducted using a Bray-Curtis distance matrix to assess the compositional dissimilarity among microbial communities. The distinct clustering of bacterial communities was observed among different treatments in both 2019 and 2020, with the first two axes explaining 30.6% and 16.8% of the total variance, respectively (Fig. 4C). Similarly, fungal communities also exhibited separate clustering, with the first two axes accounting for 28.6% and 22.1% of the total variance, respectively (Fig. 4D). These results indicate that both D and DA significantly influenced the bacterial and fungal community structure.

### Microbial community structure

**Fig 5:**
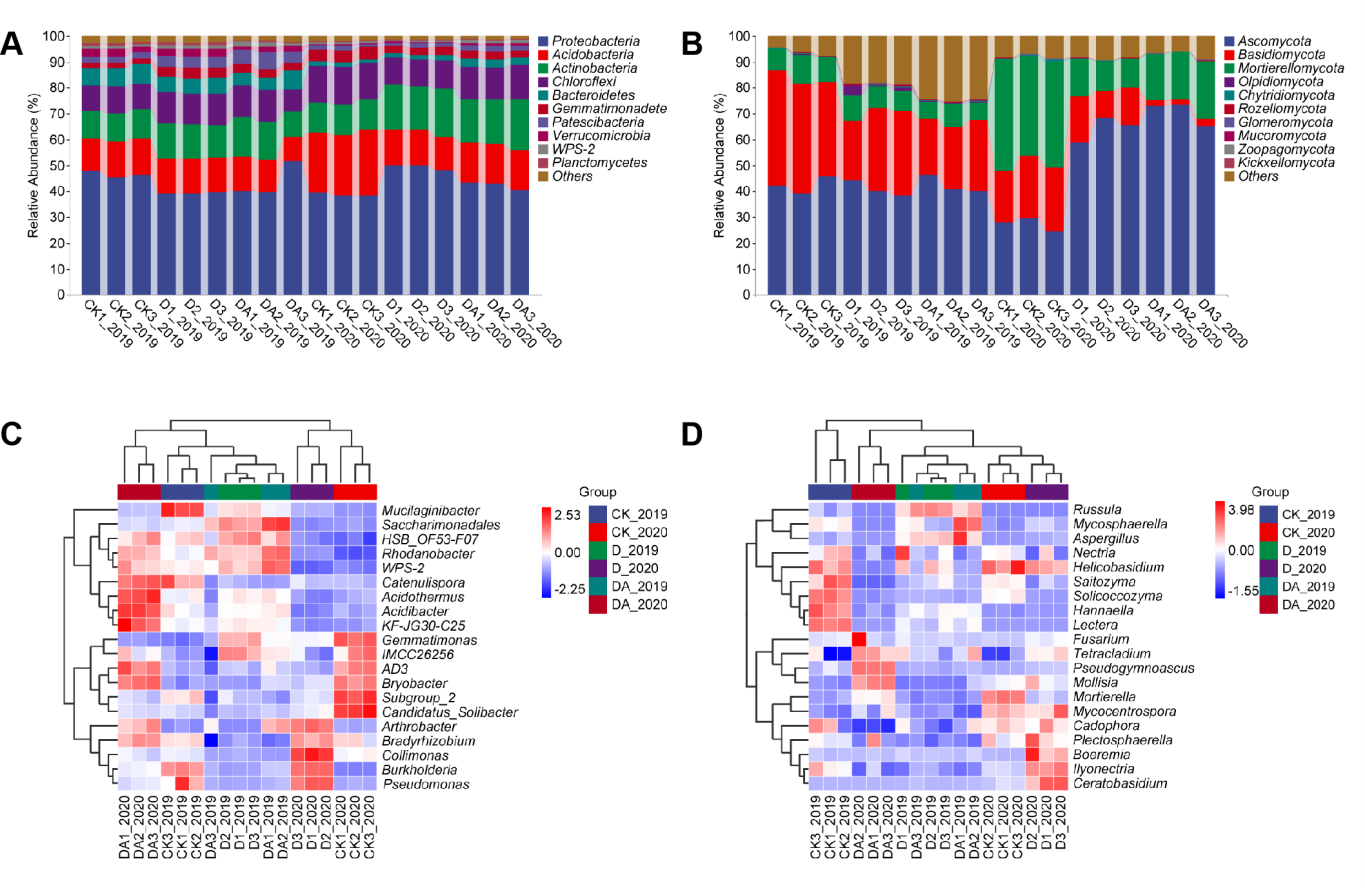
Microbial taxonomic compositions at phylum and genus levels in the rhizosphere soil of *C. tangshen*. (A) Bacterial taxonomic compositions at phylum level; (B) fungal taxonomic compositions at phylum level; (C) heatmap of the top 20 classified bacterial genera in different treatments; and (D) heatmap of the top 20 classified fungal genera in different treatments. Different colors in the heatmap denoted different microbial relative abundance, with red representing high relative abundance and blue representing low relative abundance.

At the phylum level, the top 10 predominant bacteriophyta in the rhizosphere soil of *C. tangshen* were Proteobacteria, Acidobacteria, Actinobacteria, Chloroflexi, Bacteroidetes, Gemmatimonadetes, Patescibacteria, Verrucomicrobia, WPS-2, and Planctomycetes (Fig. 5A). Moreover, these phyla constituted over 97.0% of the bacteriome, with Proteobacteria and Acidobacteria being the most dominant, comprising at least 52.7%. Notably, the proportion of Actinobacteria significantly increased in both D and DA, while Acidobacteria exhibited a contrary trend in 2020 (Table S1). The fungal communities were primarily composed of Ascomycota, Basidiomycota, Mortierellomycota, Olpidiomycota, Chytridiomycota, Rozellomycota, Glomeromycota, Mucoromycota, Zoopagomycota, and Kickxellomycota (Fig. 5B). These phyla made up more than 75.0% of the fungiome, with Ascomycota, Basidiomycota, and Mortierellomycota being the most abundant, accounting for at least 74.2%. In 2020, the abundance of Ascomycota was significantly increased in both D and DA. In contrast, the abundance of Basidiomycota and Mortierellomycota was decreased in both D and DA in 2019 and 2020 (Table S2).

At the genus level, significant differences were observed in the bacterial and fungal abundance among different treatments (Tables S3 and S4). In 2020, the relative abundance of *Catenulispora*, *Acidothermus*, *Acidibacter*, and *KF-JG30-C25* in DA was significantly higher than that in D and CK (Fig. 5C). In 2020, CK and D treatments showed significantly higher abundances of *Gemmatimonas*, *Subgroup_2*, *Candidatus_Solibacter*, *Collimonas*, *Burkholderia*, and *Pseudomonas* compared with the DA treatment. The dominant fungal genera in CK, D, and DA were distinctly different (Fig. 5D). CK had a significantly higher abundance of *Helicobasidium*, *Saitozyma*, and *Solicoccozyma* than D and DA in both 2019 and 2020. Additionally, the bacterial and fungal community structure exhibited similar characteristics between D and DA in 2019, while a similar fungal community structure was observed between CK and D in 2020.

### The functional pathways of microbial communities

**Fig 6:**
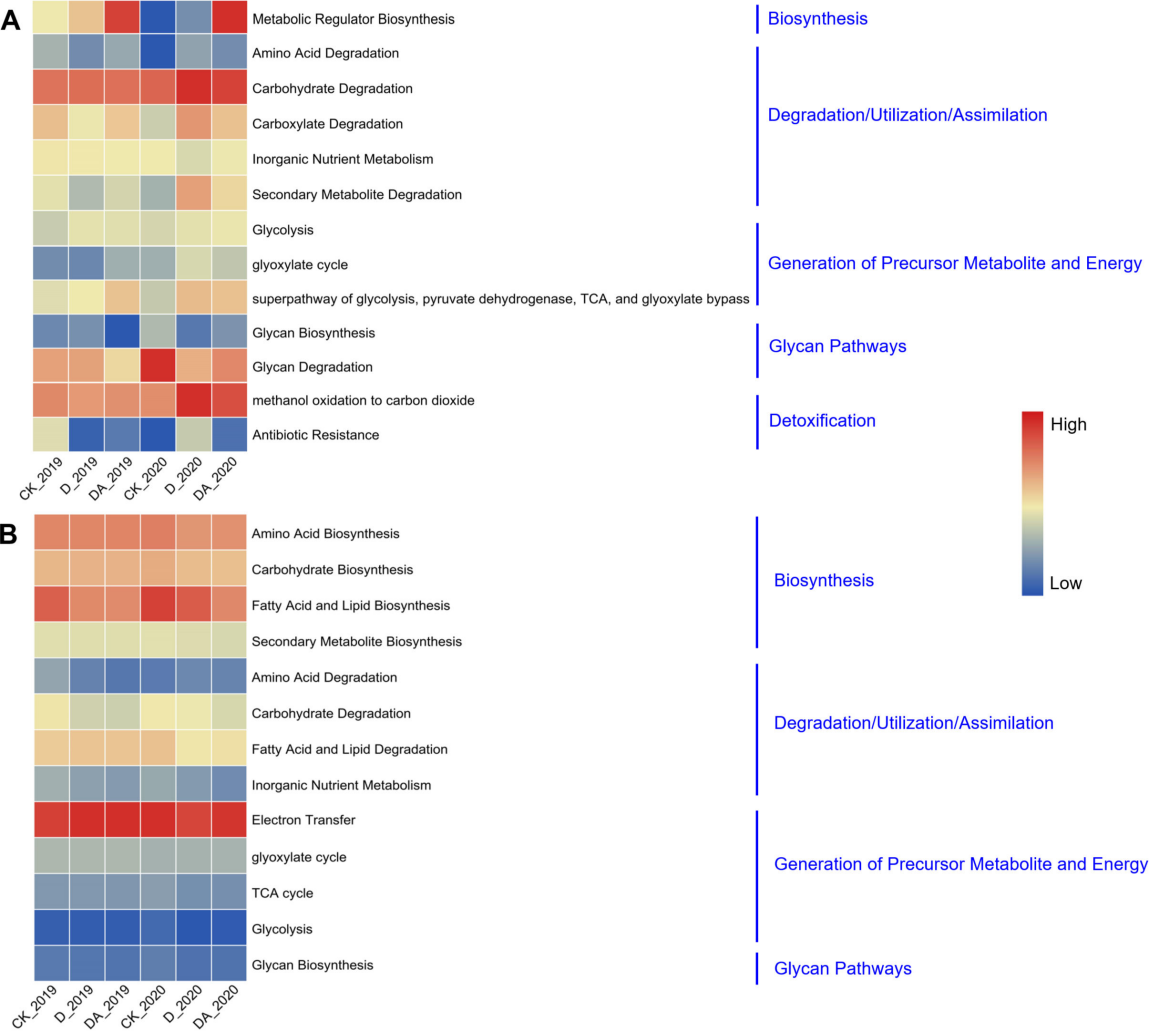
The relative abundance of level 2 KEGG pathways predicted by PICRUSt2. (A) The bacterial relative abundance of level 2 KEGG pathways predicted by PICRUSt2 and (B) the fungal relative abundance of level 2 KEGG pathways predicted by PICRUSt2.

PICRUSt2 analysis, which leverages the Kyoto Encyclopedia of Genes and Genomes (KEGG) pathway database, was conducted to predict the functional profiles of the microbial communities under different treatments (Fig. 6). The results showed that the bacterial pathways of metabolic regulator biosynthesis were significantly upregulated in both D and DA in 2019 and 2020, compared with the CK (Fig. 6A; Table S5). In 2020, the relative abundance of bacterial pathways involved in carbohydrate degradation, carboxylate degradation, secondary metabolite degradation, and methanol oxidation to carbon dioxide was notably higher in D and DA than in the CK. Conversely, the relative abundance of the glycan degradation pathway was decreased in D and DA, compared with the CK. Moreover, the relative abundance of antibiotic resistance pathways was significantly lower in both D and DA treatments in 2019 but increased notably in 2020 compared to the CK. As shown in Fig. 6B, the D and DA consistently exhibited a lower abundance of fungal pathways related to carbohydrate degradation and fatty acid and lipid degradation than the CK (Table S6).

### The correlations and interaction network analysis between microbial indices, plant indices, and soil properties

**Fig 7:**
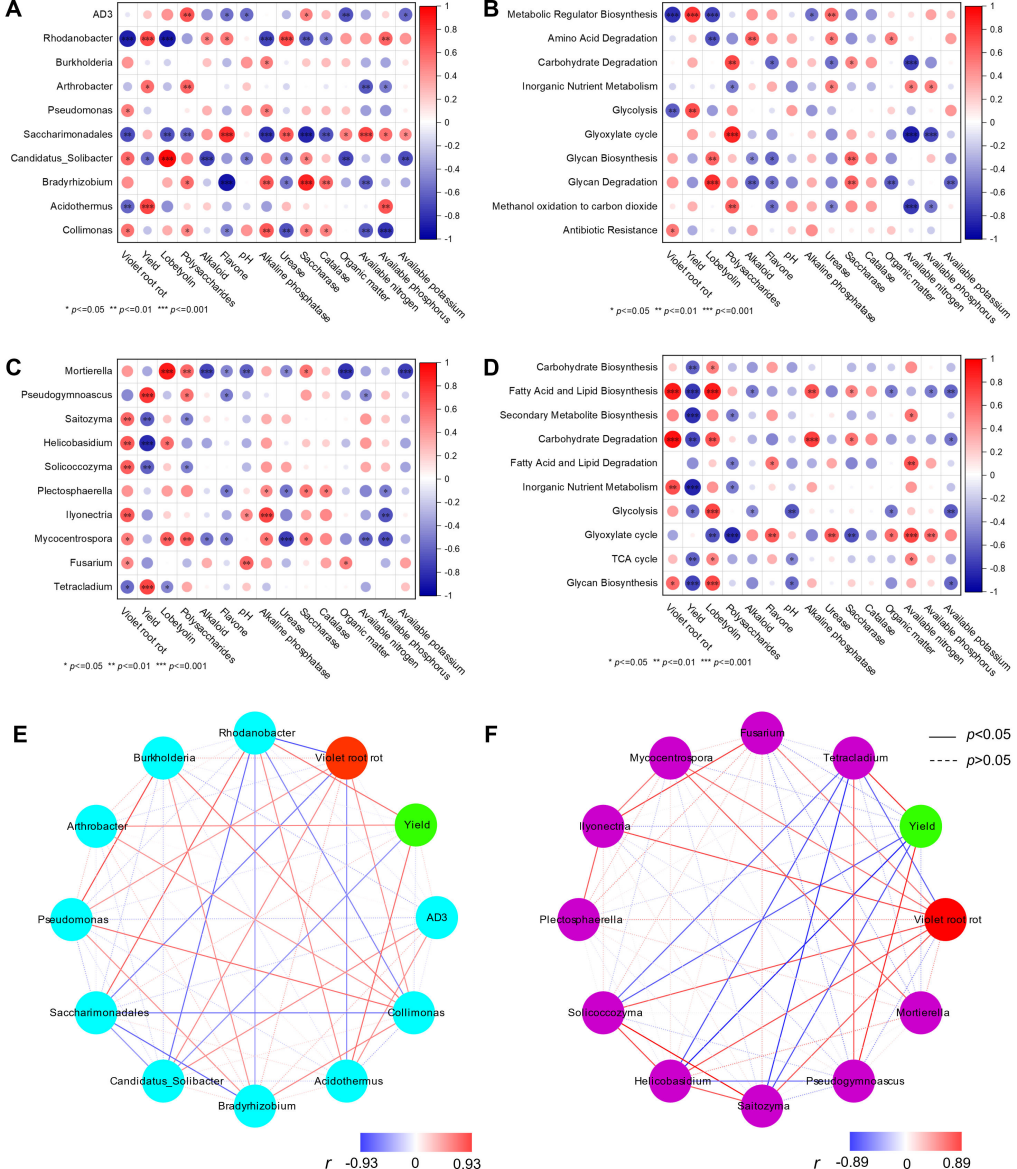
The correlation and interaction between the top 10 most abundant microbial genera, metabolic pathways, VRR incidence, plant indices, and soil properties. (A) The correlation between bacterial abundance, VRR incidence, plant indices, and soil properties; (B) the correlation between bacterial metabolic pathways, VRR incidence, plant indices, and soil properties; (C) the correlation between fungal abundance, VRR incidence, plant indices, and soil properties; (D) the correlation between fungal metabolic pathways, VRR incidence, plant indices, and soil properties; (E) the interaction network of the yield, VRR incidence, and bacterial abundance; and (F) the interaction network of the yield, VRR incidence, and fungal abundance. *, **, and *** indicate significant correlations at *P* < 0.05, *P* < 0.01, and *P* < 0.001, respectively. The water green, purple, red, and green nodes represent bacteria, fungi, VRR, and yield, respectively. Lines connecting nodes indicate positive (red) or negative (blue) relationships, with the gradient color bar representing the spectrum of Pearson’s correlation coefficients. The solid and dashed lines represent significant and non-significant correlations, respectively.

Pearson’s correlation analysis was performed to explore the relationships among the VRR, plant indices, soil properties, the top 10 most abundant microbes, and level 2 KEGG pathways. As shown in Fig. 7A, the VRR incidence demonstrated significant positive correlations with the abundance of *Pseudomonas*, *Candidatus_Solibacter*, and *Collimonas* (*P* < 0.05), and negative correlations with *Rhodanobacter*, *Saccharimonadales*, and *Acidothermus* (*P* < 0.01). The yield of *C. tangshen* was found to be positively correlated with the abundance of *Rhodanobacter*, *Arthrobacter*, and *Acidothermus*, and negatively correlated with the abundance of *Candidatus_Solibacter*. In terms of bacterial metabolic pathways, pathways such as metabolic regulator biosynthesis and glycolysis exhibited significant negative associations with the VRR incidence and significant positive associations with the yield (Fig. 7B). The medicinal component like lobetyolin was positively correlated with glycan biosynthesis and degradation pathway and negatively correlated with metabolic regulator biosynthesis and amino acid degradation pathways. The polysaccharide was positively correlated with most of the metabolic pathways, while alkaloid and flavonoid showed an opposite trend. Additionally, soil indices like urease, saccharase, organic matter, available nitrogen, and available phosphorus exhibited relatively stronger correlations with these metabolic pathways, while pH, alkaline phosphatase, catalase, and available potassium showed relatively weaker correlations with these pathways.

As shown in Fig. 7C, the VRR incidence showed significant positive correlations with the abundance of fungi like *Saitozyma*, *Helicobasidium*, *Solicoccozyma*, *Ilyonectria*, *Mycocentrospora*, and *Fusarium*, but a negative correlation with *Tetracladium*’s abundance. Conversely, the yield of *C. tangshen* showed negative relationships with the abundance of *Saitozyma*, *Helicobasidium*, and *Solicoccozyma*, and positive relationships with *Pseudogymnoascus* and *Tetracladium*. Notably, *Mortierella*, *Plectosphaerella*, and *Mycocentrospora* showed relatively stronger correlations with plant indices and soil indicators than the other fungi. Moreover, most of the fungal metabolic pathways showed positive relationships with the VRR incidence but showed negative relationships with the yield (Fig. 7D). Interestingly, nearly all the fungal metabolic pathways exhibited positive relationships with the lobetyolin. Furthermore, the fungal metabolic pathways showed relatively weak relationships with the plant indices and soil indicators, except pathways like fatty acid and lipid biosynthesis, carbohydrate degradation, and the glyoxylate cycle.

Interaction network analysis was conducted to explore the synergistic and antagonistic relationships between microbial communities and their impact on the yield and VRR incidence. Most of the bacteria exhibited negative correlations with the VRR incidence and positive correlations with the yield (Fig. 7E). Specifically, the synergistic effects of *Pseudomonas*, *Candidatus_Solibacter*, and *Collimonas* may negatively contribute to the VRR incidence, while the positive effects of *Rhodanobacter*, *Saccharimonadales*, and *Acidothermus* may enhance the yield. In contrast, most of the fungi showed positive correlations with VRR incidence and negative correlations with the yield (Fig. 7F). Overall, the synergies of *Saitozyma*, *Helicobasidium*, *Solicoccozyma*, *Ilyonectria*, *Mycocentrospora*, and *Fusarium* may be pivotal in VRR infection. It was noteworthy that *Tetracladium*, which correlated positively with the yield and negatively with VRR incidence, may be a beneficial species for both VRR control and *C. tangshen* growth.

## DISCUSSION

### Co-application of dazomet and azoxystrobin affected the yield, violet root rot incidence, medicinal component level, and rhizosphere soil properties

VRR is a pervasive soil-borne disease that poses a significant challenge to the cultivation of *C. tangshen*. It usually takes several months or even a year for *C. tangshen* from infection with VRR to death. Previous studies have shown that soil fumigation with dazomet can substantially boost crop yields and decrease the incidence of soil-borne disease (10, 17, 18). Consistent with these findings, our study demonstrated that both soil fumigation (D) alone and in combination with azoxystrobin (DA) significantly increased the yield and reduced the incidence of VRR in *C. tangshen* under continuous cropping conditions (Fig. 1). Notably, the combined application of dazomet and azoxystrobin (DA) completely prevented the occurrence of VRR in both 2019 and 2020, a result not achieved by dazomet alone (D). These results suggested that co-application of dazomet and azoxystrobin was more effective in controlling VRR than soil fumigation alone, and the control effect lasted for at least 2 years. Medicinal plants typically accumulate higher levels of secondary metabolites under stress to adapt to environmental changes, whereas under favorable conditions, they prioritize primary metabolite production for biomass accumulation (19). In this study, DA reduced lobetyolin content but increased polysaccharide levels in both 2019 and 2020, suggesting that continuous cropping stress may enhance lobetyolin accumulation, whereas DA-mediated stress alleviation favors polysaccharide production (20).

Soil is a pivotal environment that supplies a plethora of essential nutrients necessary for plants (21). Soil fumigation has been shown to significantly alter the soil properties (22). In our study, soil enzyme activities were generally downregulated in 2019 and rebounded in 2020, aligning with the fluctuating trends of the top 20 bacterial and fungal abundance (Tables S3 and S4). These results suggested that the top 20 microbes may exert a crucial role in soil nutrient metabolism. In 2020, both D and DA treatments significantly increased the soil organic matter and available phosphorus content, which likely provided sufficient nutrients for the growth of *C. tangshen*. This may partially explain the significant yield enhancement observed under these treatments. Soil pH can directly or indirectly affect the abundance of microbial populations, thereby influencing soil nutrient metabolism (23, 24). Typically, healthy non-continuous cropping soils maintain a pH above 5.0, whereas after years of monoculture cultivation, the soil pH of continuous cropping plots drops below 4.7 (Fig. 3). Previous studies have revealed that soil pH is positively associated with the abundance of beneficial microbes (pathogen-suppressive microbes) and negatively associated with soil pathogens (25, 26). Interestingly, our observations revealed that soil pH correlated positively with the abundance of some pathogens, such as *Ilyonectria* and *Fusarium*, and negatively with the abundance of potential beneficial microbes like *Mortierella*. These findings underscore the intricate relationships between soil pH and microorganisms and highlight the necessity for further research to elucidate these interactions.

### Co-application of dazomet and azoxystrobin altered the microbial diversity, community structure, and functional pathway

Soil microbial diversity plays a pivotal role in plant growth, as microbes play an important role in soil nutrient cycling, soil structure maintenance, and soil-borne disease control (18, 27, 28). In our study, DA significantly decreased bacterial Shannon indices in both 2019 and 2020, indicating that bacteria showed strong sensitivity to dazomet fumigation combined with azoxystrobin application. However, the co-application of dazomet and azoxystrobin (DA) initially upregulated fungal Shannon and Simpson indices in 2019, while downregulated chao1 and Shannon indices in 2020. We hypothesized that the non-selective nature of dazomet resulted in the elimination of dominant fungi in 2019, which may temporarily increase fungal α-diversity indices. As certain fungi re-established their dominance in 2020, a subsequent decrease in α-diversity indices was observed. Additionally, the β-diversities in D and DA were found to be significantly different from the control, with a notable difference also observed between D and DA. Thus, considering the results of both α-diversity and β-diversity, we speculated that D and DA can significantly restructure the microbial community, thereby promoting plant growth and decreasing pathogen invasion.

Soil microbial community structure plays a pivotal role in soil biological and chemical cycling, directly or indirectly influencing plant growth (27, 29). Extensive research has shown that soil fumigation can alter the microbial community structure in the rhizosphere soil of crops under continuous cropping systems (10, 17, 27). In the present work, both the D and DA treatments affected the abundance of certain microbes, leading to changes in the microbial community structure at both the phylum and genus levels (Fig. 5). These results suggested that specific microbes exhibited unique sensitivities to fumigation or fungicide application, aligning with previous studies which showed the impact of soil fumigation on rhizosphere microbial community structure (10, 30). Notably, VRR incidence showed significant positive correlations with most of the top 10 fungal genera, while it exhibited significant negative correlations with three bacterial genera (Fig. 7). Hence, we speculated that D and DA induced a shift in the rhizosphere microbial community, potentially leading to the reassembly of a disease-suppressive community from one initially dominated by pathogens, which may be crucial for controlling VRR. This hypothesis was supported by the results of principal coordinate analysis (PCoA) (Fig. 4) and lower disease incidence in D and DA (Fig. 1).

The changes in microbial community structure induced by soil fumigation can significantly affect microbial interactions and functional pathways (31, 32). In this study, the DA significantly upregulated the bacterial pathways of metabolic regulator biosynthesis, carbohydrate degradation, carboxylate degradation, secondary metabolite degradation, and methanol oxidation to carbon dioxide, while significantly downregulated the fungal pathways of carbohydrate degradation and fatty acid and lipid degradation, indicating that DA has a significant effect on microbial metabolic activities. Moreover, most of the fungal metabolic pathways showed positive relationships with the VRR incidence but exhibited negative relationships with yield, indicating that fungal metabolic activities may promote the occurrence of VRR in *C. tangshen*.

### Violet root rot associated with pathogen-suppressive microbes and soil-borne pathogens

Numerous studies have shown that the occurrence of soil-borne disease is linked to a decline in pathogen-suppressive microbes and an accumulation of soil-borne pathogens (25, 33). Potential pathogen-suppressive microbes, such as *Bacillus*, *Pseudomonas*, *Burkholderia*, *Bradyrhizobium*, and *Mortierella*, are crucial for promoting plant growth and protecting crops from soil-borne diseases (10, 27, 34). Conversely, soil-borne pathogens, including *Helicobasidium*, *Fusarium*, *Ilyonectria*, and *Mycocentrospora*, can cause diseases like root rot, damping-off, and wilt, which significantly impede crop growth, survival, and yield (27, 35–37). In this study, the DA significantly decreased the abundance of certain soil-borne pathogens like *Helicobasidium*, *Fusarium*, *Ilyonectria*, *Mycocentrospora*, *Saitozyma*, and *Solicoccozyma*, while increasing the abundance of some potential pathogen-suppressive microbes like *Rhodanobacter*, *Saccharimonadales*, *Acidothermus*, and *Tetracladium*. Moreover, the abundance of *Helicobasidium* was positively correlated with the abundance of *Saitozyma* and *Solicoccozyma*, while it showed a negative correlation with the abundance of *Tetracladium*. We speculated that prior to soil fumigation, the dominant microbes like *Helicobasidium*, *Fusarium*, and *Ilyonectria* may suppress the growth of other microbes, resulting in relatively lower abundance of pathogen-suppressive microbes. After fumigation, the dominant pathogens are largely eradicated, releasing more available niches for other microbes. Pathogen-suppressive microbes gained a competitive advantage in occupying these niches, enabling them to proliferate rapidly in the rhizosphere soil (30). Consequently, the DA treatment resulted in a reduced abundance of soil-borne pathogens and an enrichment of pathogen-suppressive microbes.

Previous studies showed that *Rhodanobacter*, *Saccharimonadales*, and *Acidothermus* played pivotal roles in the control of plant diseases (38–40). In this study, the VRR incidence of *C. tangshen* was negatively correlated with bacteria such as *Rhodanobacter*, *Saccharimonadales*, and *Acidothermus* (Fig. 7), indicating that these microbes may play a critical role in controlling VRR. Additionally, the VRR incidence was negatively correlated with *Tetracladium*, indicating that *Tetracladium* may be a potential beneficial fungus in controlling *C. tangshen* VRR, as *Tetracladium setigerum* possesses bioactivity against various microbial pathogens (41). However, the potential pathogen-suppressive microbes like *Burkholderia* and *Bradyrhizobium* did not exhibit a significant relationship with the incidence of VRR. We supposed that these microbes may have variable effects in different soil micro-environments. However, the positive correlation between *Pseudomonas* and VRR incidence was contradictory to the earlier studies, which reported that *Pseudomonas* could mitigate soil-borne diseases and environmental stress in plants (42, 43). This discrepancy may be attributed to the distinct ecological functions among different Pseudomonas species.

**Fig 8:**
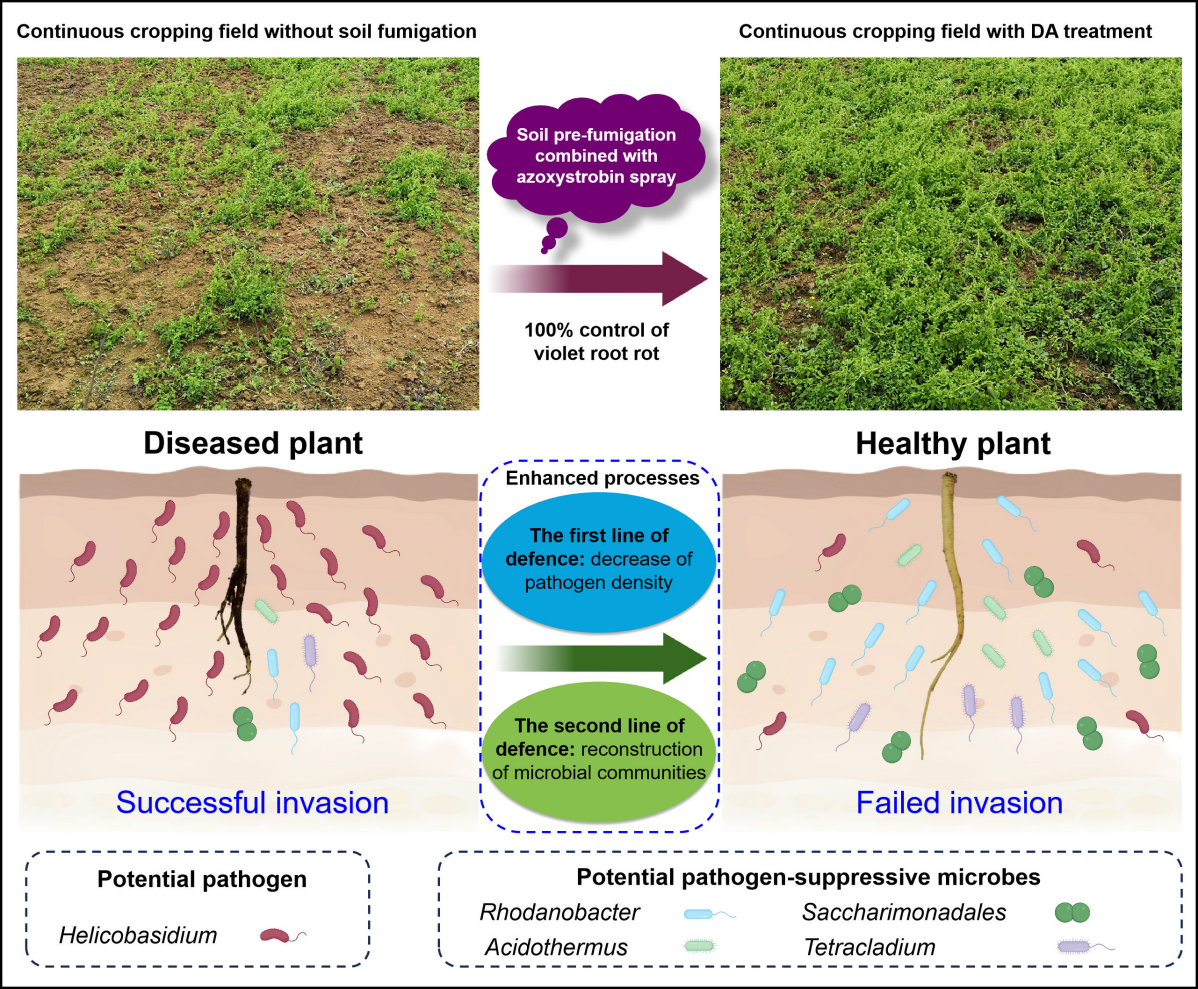
A hypothetical model showing DA treatment-induced changes in *C. tangshen* growth and soil microbial community structure under a continuous cropping system. The decrease of pathogen density is defined as “the first line of defense,” and the reconstruction of microbial communities is defined as “the second line of defense.”

The abundance of potential pathogen *Helicobasidium* was significantly decreased after soil fumigation combined with azoxystrobin application (Table S4), which was defined as “the first line of defense” (Fig. 8), and may directly contribute to the substantial decrease in VRR incidence. This speculation was supported by the positive correlation between *Helicobasidium* abundance and VRR incidence, and the negative correlation between *Helicobasidium* abundance and yield (Fig. 7). Moreover, we speculated that the VRR incidence was indirectly reduced by DA-induced enrichment of *Rhodanobacter*, *Saccharimonadales*, *Acidothermus*, and *Tetracladium*, which was defined as “the second line of defense” (microbial community reconstruction, Fig. 8), since these pathogen-suppressive microbes may reduce pathogen infection by competitively occupying more ecological niches in the rhizosphere (25). This hypothesis was corroborated by both interaction network analysis (Fig. 7) and the changes in microbial community structure following DA treatment (Tables S3 and S4). Unfortunately, despite extensive efforts to isolate *Helicobasidium* pathogens from infected plants, we failed to obtain pure cultures in this study, highlighting challenges in culturing this VRR pathogen. Further methodological refinements will be pursued to achieve pure cultures.

### Conclusions

This study demonstrated that co-application of dazomet and azoxystrobin (DA) effectively reduced the VRR incidence and increased the yield of *C. tangshen* under a continuous cropping system. Furthermore, DA treatment significantly decreased the abundance of potential pathogen *Helicobasidium*, while increasing the abundance of potential pathogen-suppressive microbes like *Rhodanobacter*, *Saccharimonadales*, *Acidothermus*, and *Tetracladium*. Notably, VRR incidence exhibited a significant positive correlation with *Helicobasidium* abundance and a negative correlation with the abundance of these potential pathogen-suppressive microbes, suggesting that DA treatment may control the VRR by decreasing pathogen density and reconstructing microbial communities. Additionally, the DA treatment can also change partial biochemical properties of the soil, including pH value, enzyme activities, and nutrient levels. This study provides an effective solution for controlling VRR in *C. tangshen*, with strong application potential and excellent prospects for promotion.

## MATERIALS AND METHODS

### Site description and experimental design

Two-year field trials were conducted at a demonstration base for continuous cropping obstacle control in Xintian Village (30°32′ 28″ N, 109°12′ 35″ E, 1,872 m), Banqiao Town, Enshi City, Hubei Province, China. The experimental site had a history of continuous *C. tangshen* cultivation for the past 6 years on a plot characterized by its typical acidic soil (4.72 of pH, 43.0 g/kg of organic matter, 2.3 g/kg of total nitrogen, 1.1 g/kg of total phosphorus, 21.6 g/kg of total potassium, 309.7 mg/kg of alkali-hydrolyzed nitrogen, 37.5 mg/kg of available phosphorus, and 234.6 mg/kg of available potassium).

This study utilized 1-year-old *C. tangshen* seedlings as test materials. A field experiment was carried out in November 2018, involving three different treatments. Namely, CK: continuous cropping of *C. tangshen* without soil fumigation; D: soil pre-fumigation with 300 kg·hm^−2^ dazomet (Lanfeng Biochemical, China); DA: soil pre-fumigation with dazomet (D), followed by soaking *C. tangshen* roots in a 1,000-fold dilution of azoxystrobin (Jiangsu Frey Agrochemicals, China) for 30 minutes before planting, and three foliar sprays of the same azoxystrobin dilution every 30 days once the seedlings reached 10 cm in height. In detail, soil fumigation was performed approximately 1 month prior to the *C. tangshen* seedling transplanting. To begin, the continuous cropping soil was watered to adjust the relative soil humidity to 70.0%, after which dazomet was applied. The experimental plots were then covered with plastic films for fumigation, which lasted for 25 days. Afterward, the soil was tilled to allow for gas venting. Prior to transplanting, a safety verification was performed using a germination test with Chinese cabbage seeds. A compound fertilizer (nitrogen:phosphorus pentoxide:potassium oxide = 15:15:15) was applied at a rate of 750 kg·hm^−2^. This experiment followed a randomized block design with three replicates (plots) per treatment. Each plot was 24 m^2^ (1.2 m × 20 m), with plants spaced at 25 cm × 10 cm.

### Sample collection and preparation

In this research, the experimental plots were harvested in two separate phases: the first harvest occurred in September 2019, followed by the final harvest in September 2020. The five-point random sampling method was employed to collect *C. tangshen* roots and corresponding rhizosphere soil. Each sampling point covered an area of 1 m^2^, and the roots and rhizosphere soils from five points were mixed to form a composite root and soil sample, respectively. The iron-toothed rake was used to carefully unearth the roots of *C. tangshen*, followed by shaking to remove large clumps of surface soil. The remaining soil attached to the roots was then gently brushed off using a soft-bristle brush, which was designated as the rhizosphere soil. The roots were meticulously washed with distilled water and subsequently dried at 45°C until they reached a constant weight. For diseased roots with visible pathogenic fungal mycelium, a stiff-bristled brush was used to remove the mycelium during the cleaning process. Once dried, the roots were ground into powder using a grinder and stored in sterile, self-sealing plastic bags for subsequent determination of medicinal components. The collected rhizosphere soil was divided into two parts. One part was immediately frozen at −80°C for microbiome analysis, while the other part was air-dried at room temperature for subsequent assessment of enzyme activities and physicochemical properties.

### Sample measurement

The yield was determined by measuring the fresh weight of the harvested *C. tangshen* roots within five points (about 40 plants per point) mentioned above. The existing *C. tangshen* roots sampled from five points in one plot were mixed as one sample for yield calculation and VRR investigation. The VRR incidence was calculated using the following equation: disease incidence (%) = (number of infected plants/total number of surveys) × 100. Infection is confirmed when the pathogen’s mycelium becomes visible on the root surface of *C. tangshen*. The key medicinal components of *C. tangshen,* like polysaccharides, alkaloids, and flavonoids, were quantified by UV-Vis spectrophotometry according to the previous studies (44–46). Determination of lobetyolin was performed by high-performance liquid chromatography using an Agilent 1260 Infinity LC system (45). Specifically, the standard curve (*y* =  746.55x + 11.81, r^2^ = 0.9992) was established using the lobetyolin standard sample with purity >98.0%. The formula for calculating lobetyolin content was Lobetyolin content (mg·kg^−1^) = (PA − 11.81)/(746.55 × SDW). PA and SDW denote peak area and sample dry weight, respectively.

Soil samples were prepared by mixing them with distilled water at a 1:2.5 (soil to water) ratio, agitated for 30 minutes to release CO_2_, and then the soil pH was measured using a pH meter (innoLab 20P, Prima, UK). The activities of key soil enzymes like alkaline phosphatase, urease, saccharase, and catalase, as well as soil nutrients including organic matter, alkali-hydrolyzed nitrogen, available phosphorus, and available potassium, were determined according to the Soil Chemistry Analysis Handbook (47).

### Amplicon sequencing and bioinformatic analysis

All molecular biology operations, including DNA extraction, PCR amplification, and amplicon sequencing, were carried out by Shanghai Personal Biotechnology Co., Ltd. in Shanghai, China. The 16S rRNA gene for bacteria and the ITS gene for fungi were specifically targeted using the primer pairs 338F/806R (5′-ACTCCTACGGGAGGCAGCA-3′/5′-GGACTACHVGGGTWTCTAAT-3′) and ITS5F/ITS1R (5′-GGAAGTAAAAGTCGTAACAAGG-3′/5′-GCTGCGTTCTTCATCGATGC-3′), respectively (48). PCR amplification was conducted using a thermocycler PCR system (GeneAmp 9700, ABI, USA). Purified amplicons were pooled in equimolar concentration of 10 ng·μL^−1^ and paired-end sequenced (2 × 300) on an Illumina MiSeq platform (Illumina, San Diego, USA). Finally, all raw sequences have been uploaded to the NCBI Sequence Read Archive database with a BioProject ID: PRJNA1133669 (https://www.ncbi.nlm.nih.gov/).

The low-quality (<0.001%) sequences were discarded according to our previous research (20), and the high-quality sequences were assigned to operational taxonomic units (OTUs) at a 97.0% nucleotide similarity. Taxonomy annotation of the OTUs was performed based on the Naive Bayes classifier in QIIME2 using the SILVA database with a confidence threshold of 70.0% (27). The potential functional pathways of the microbial communities were predicted by PICRUSt2. Additionally, the relationships between microbial communities, yield, and the incidence of VRR were explored through Pearson correlation coefficients. The interaction network was visualized using Cytoscape (version 3.8.2), providing a graphical representation of these relationships.

### Statistical analysis

Significant differences among different treatments were determined using one-way analysis of variance and Duncan’s multiple range test, as analyzed by SPSS 20.0 software, with a significance level set at *P* < 0.05. Additionally, Pearson’s correlation analysis was employed to explore the relationships between yield, disease incidence, medicinal components, soil properties, and microbial abundance. The results presented in the histogram are depicted as the mean values derived from three replicates, accompanied by their corresponding standard deviations. The plotting of bar charts was performed using GraphPad Prism 8. For the visualization of microbial community data, PCoA, microbial diversity, and heatmap were performed using R (version 4.4.1) or TBtools software (Version 2.096) (49).

## SUPPLEMENTAL MATERIAL

Table S1 spectrum.01088-25-s0001.xls Bacteria abundance at the phylum level.

Table S2 spectrum.01088-25-s0002.xls Fungi abundance at the phylum level.

Table S3 spectrum.01088-25-s0003.xls Top 20 bacteria abundance at the genus level.

Table S4 spectrum.01088-25-s0004.xls Top 20 fungi abundance at the genus level.

Table S5 spectrum.01088-25-s0005.xls The relative abundance of bacterial level 2 KEGG pathways.

Table S6 spectrum.01088-25-s0006.xls The relative abundance of fungal level 2 KEGG pathways.

ASM does not own the copyrights to Supplemental Material that may be linked to, or accessed through, an article. The authors have granted ASM a non-exclusive, world-wide license to publish the Supplemental Material files. Please contact the corresponding author directly for reuse.
